# A novel machine learning model based on ubiquitin-related gene pairs and clinical features to predict prognosis and treatment effect in colon adenocarcinoma

**DOI:** 10.1186/s40001-023-00993-z

**Published:** 2023-01-21

**Authors:** Liping Liang, Le Liu, Shijie Mai, Ye Chen

**Affiliations:** 1 Department of Gastroenterology, State Key Laboratory of Organ Failure Research, Guangdong Provincial Key Laboratory of Gastroenterology, Nanfang Hospital, Southern Medical University, Guangzhou, 510515 China; 2 Department of Gastroenterology, Integrated Clinical Microecology Center, Shenzhen Hospital, Southern Medical University, 1333 New Lake Road, Shenzhen, 518100 China; 3 Department of Thoracic Surgery, Nanfang Hospital, Southern Medical University, Guangzhou, 510515 China

**Keywords:** Colon adenocarcinoma, Prognostic signature, Tumor immune microenvironment

## Abstract

**Background:**

Ubiquitin and ubiquitin-like (UB/UBL) conjugations are essential post-translational modifications that contribute to cancer onset and advancement. In colon adenocarcinoma (COAD), nonetheless, the biological role, as well as the clinical value of ubiquitin-related genes (URGs), is unclear. The current study sought to design and verify a ubiquitin-related gene pairs (URGPs)-related prognostic signature for predicting COAD prognoses.

**Methods:**

Using univariate, least absolute shrinkage and selection operator (LASSO), and multivariate Cox regression, URGP's predictive signature was discovered. Signatures differentiated high-risk and low-risk patients. ROC and Kaplan–Meier assessed URGPs' signature. Gene set enrichment analysis (GSEA) examined biological nomogram enrichment. Chemotherapy and tumor immune microenvironment were also studied.

**Results:**

The predictive signature used six URGPs. High-risk patients had a worse prognosis than low-risk patients, according to Kaplan–Meier. After adjusting for other clinical characteristics, the URGPs signature could reliably predict COAD patients. In the low-risk group, we found higher amounts of invading CD4 memory-activated T cells, follicular helper T cells, macrophages, and resting dendritic cells. Moreover, low-risk group had higher immune checkpoint-related gene expression and chemosensitivity.

**Conclusion:**

Our research developed a nomogram and a URGPs prognostic signature to predict COAD prognosis, which may aid in patient risk stratification and offer an effective evaluation method of individualized treatment in clinical settings.

**Supplementary Information:**

The online version contains supplementary material available at 10.1186/s40001-023-00993-z.

## Introduction

Colon cancer is the most prevalent malignancy affecting the gastrointestinal tract, with an estimated 101,420 newly diagnosed cases and 51,020 deaths in the United States in 2019 [1]. Colon adenocarcinoma (COAD) accounts for over 80% of all colon malignancies, with sarcomas and squamous cell carcinomas responsible for the remaining 20% [1, 2]. COAD is typically treated with surgery, chemotherapy, radiation therapy, immunotherapy, and other treatments. Cass et al. found that following complete primary resection, 37% of patients suffered a local recurrence and distant metastases, with the most prevalent contributor to mortality within 5 years being local recurrence in the absence of clinical indication of distant metastases [3]. Chemotherapy can be administered as adjuvant therapy after surgery or as neoadjuvant therapy before surgery in advanced COAD patients to help decrease the tumor. Despite this, 40–50% of advanced COAD patients die as a result of disease recurrence or metastasis [4]. As a result, a distinct COAD prediction signature is critical, with the potential to identify new therapeutic targets and prognosis markers.

Tumor cells can effectively change their microenvironment by producing a variety of chemokines, cytokines, and other substances. Immunotherapy is commonly utilized for the treatment of malignancies in humans. Over the last several decades, many inhibitory receptors have been shown to perform an integral function in dampening anti-tumor immune responses. Included among these are programmed death-ligand 1 (PD-L1), cytotoxic T lymphocyte-associated antigen-4 (CTLA4), and programmed cell death protein-1 (PD-1). COAD-related early treatment and first-line therapy include immune checkpoint blockade (ICB) to serve a greater number of patients. Other immune-associated indicators have also been discovered, opening the way for more efficacious immunotherapy and demonstrating immunotherapy's promise as a COAD treatment regime.

Ubiquitin and ubiquitin-like (UB/UBL) conjugations are essential post-translation modifications that are required for almost all biological activities and pathways, particularly protein breakdown and turnover, DNA damage repair, and cell cycle, as well as intercellular signal transmission. Ubiquitin is a protein that is evolutionary conserved and is known to modify proteins post-translationally either for degradation or non-degradative signaling. In addition, it is covalently linked to lysine residues sequentially by 3 enzymes, namely ubiquitin-activating enzymes (E1s), ubiquitin-conjugating enzymes (E2s), and ubiquitin-protein ligases (E3s). UB's C-terminus is initially triggered by an E1 activating enzyme before being transported to the catalytic domain of an E2 conjugating enzyme. Furthermore, an E3 ubiquitin ligase connects the target protein and the E2-ubiquitin intermediate to act as a catalyst for the creation of an isopeptide bond between the UB C-terminal glycine and substrate lysine [7, 8]. Deubiquitinases (DUBs), which are Ub-specific proteases, act as catalysts for the elimination of UB from substrates since it is a reversible post-translational modification. There are roughly 100 distinct DUBs in humans, which may be roughly divided into seven structurally diverse superfamilies. DUBs regulate key cellular functions by cleaving UB bound to substrates or inside UB chains, acting as either switch to eliminate UB signals or rheostats to fine-tune the amount and kind of ubiquitylation [9, 10]. Furthermore, proteins with ubiquitin-like domains (ULDs) and ubiquitin-binding domains (UBDs) perform an integral function in ubiquitination regulation [9, 11]. Numerous human illnesses, including cancer and neurodegenerative disorder, have been linked to protein ubiquitination dysfunction, according to research [12]. However, no research has looked into the link between ubiquitin-related genes (URGs) and COAD patients' prognosis.

In this research, we analyzed COAD patients' gene expression patterns and clinicopathological data to develop a unique 6-ubiquitin-related gene pairs (URGPs) profile for predicting prognoses and immune responses. Our URGPs signature will offer an insightful comprehension of the tumor immune milieu and the treatment efficacy in COAD.

## Materials and methods

### Acquisition and processing of data

The Cancer Genome Atlas (TCGA, https://portal.gdc.cancer.gov/) was retrieved to acquire the transcriptomic data as well as the relevant clinicopathological data, which comprised 367 tumors tissues. The transcriptomic data were then subjected to background adjustment and normalization utilizing a style of fragments per kilobase million (FPKM) [13]. When any expression values of the genes were 0 occurring in over 50% of the samples, they would be deleted. In cases where genes were duplicated, an analysis of their average expression levels was performed. Patients who lacked matching RNA-seq data or clinical data, as well as those who had a survival duration of fewer than 30 days, were not included in the research. The TCGA database access guidelines were followed for all of the obtained profiles. Additional file 1: Table S1 illustrates the comprehensive clinical data from the aforementioned datasets. Since the data for this study were acquired from publicly available sources, there was no need to acquire permission from the local ethics committee.

The iUUCD 2.0 database (http://iuucd.biocuckoo.org/) yielded a total of 807 URGs (Additional file 2: Table S2) [14]. By screening the TCGA-COAD dataset, we retrieved 782 URGs that had readily accessible mRNA expression. URGs having a median absolute deviation (MAD) of less than 0.5 were eliminated to guarantee accurate prediction.

To generate an index for every URGP present in each sample, a paired comparison translation was carried out between the potential URG expression values. When the expression levels of the former URG were found to be greater than that of the latter URG, then the URGP was allocated a value of 1; otherwise, it was given a value of 0. URGPs were retained if their gene ratios (1/0 or 0/1) were greater than 0.2 and less than 0.8.

### Weighted gene co-expression network analysis (WGCNA)

The R package 'WGCNA' was used in our study to generate and analyze a co-expression network for URGs. Using average linkage and Pearson's correlation coefficients, the COAD samples were clustered. To build the co-expression gene network, a power of 4 was chosen as the soft-threshold parameter. Using the adjacency matrix, we then computed a topological overlap matrix (TOM). TOM dissimilarity was assessed in order to perform module partition analysis. To classify genes with similar expression patterns, a hierarchical clustering tree of genes was constructed. Following that, we used the Dynamic Tree Cut algorithm to obtain the network modules by cutting the tree's branches. For further investigation, the most important genes in each module were chosen. There are two limiting factors in this step: gene significance (GS) and module membership (MM) [15].

### Development of a risk model to analyze the risk score

First, the prognostic-associated URGPs were detected by conducting a univariate Cox regression analysis. Then, with the help of the "glmnet" package in R software (version 4.0.4), the least absolute shrinkage, and selection operator (LASSO) Cox regression analysis was performed to determine the significant prognostic URGPs. Following that, a multivariate stepwise Cox regression proportional hazards regression model was developed to additionally identify URGPs and for model optimization [15]. Ultimately, a risk score equation was derived by combining the regression coefficients from the multivariate Cox analysis with the URGPs expression values that corresponded to those coefficients. The following is the risk score equation: Risk score = (exp URGP1 × coef URGP1) + (exp URGP2 × coef URGP2) + … + (exp URGPn × coef URGPn). Herein, "exp" refers to the expression of genes that have been optimized, while "coef" refers to the derived multivariate Cox regression coefficients.

Besides, Pearson analysis was used to identify transcription factors (TFs) and enhancer RNAs (eRNAs) of interest (|Pearson cor|> 0.4 and *p* < 0.001), and a Sankey diagram was constructed to examine the potential relationship between URGs and TFs/eRNAs [15]. When conducting a survival analysis, it is a usual practice to stratify the data premised on both the median value and the optimum threshold value. In this case, we used the training dataset's median risk score as the threshold value for the training and validation data sets. Kaplan–Meier curve analysis and area under receiver operating characteristics (ROC) curve analysis were conducted utilizing the "survival" and the "timeROC" packages, respectively, to examine the prognostic significance of the URGPs-based signature. In addition to this, the testing dataset was applied to confirm the robustness and accuracy of the URGPs signature. *p* values of less than 0.05 were used as the criterion for determining statistical significance.

### Nomogram development and validation

Univariate and multivariate Cox analyses were utilized in the TCGA dataset to detect independent prognostic characteristics that integrated the URGPs signature and clinical-pathological features. Following that, a predictive nomogram was designed for the prediction of COAD patients' overall survival (OS) over one, three, and five years. To determine the nomogram's predictive accuracy, calibration plots were generated. The performance of the nomogram was subsequently evaluated using Kaplan–Meier curve analysis, the area under the curve (AUC) of the ROC curve, and the concordance index (C-index) (generated with the aid of the "survcomp" package). In this study, *p* values of < 0.05 were set as the criterion for determining statistical significance.

### Gene set enrichment analysis (GSEA) for Kyoto Encyclopedia of Genes and Genomes (KEGG)

GSEA is a method for determining if gene sets, as opposed to individual genes, exhibit variations across biological status groups and for confirming the enrichment of gene sets within a clinical group [18]. The gene sets have already been established based on earlier studies and function annotations. Then, we selected gene sets from the KEGG pathway, which offers a compilation of pathway maps showing the molecular interactions, reactions, and network relationship [19]. We carried out GSEA-KEGG analyses and generated enrichment plots in the TCGA cohort by employing "c2.cp.kegg.v7.4.entrez.gmt" and the R packages "clusterProfiler" with the filtering criteria of *p*-value less than 0.05 and *q*-value less than 0.05.

### Calculation of tumor microenvironment (TME) cell infiltration and correlation of risk score with microsatellite instability (MSI) and tumor mutation burden (TMB)

To determine the proportions of 22 distinct kinds of infiltrating immune cells in each of the COAD samples that were examined, the CIBERSORT algorithm was utilized [20]. The cutoff value that we utilized to determine whether or not a prediction of immune cell infiltration was accurate was set at *p*-value less than 0.05. Only the samples that met this criterion were eligible for further study. The "maftools" package was used to evaluate mutated genes in the various risk categories. The "limma" and "ggpubr" packages were used to investigate the relationship between TMB and URGPs-associated prognostic genotyping. Additionally, we compared the connections between MSI and the high-risk and low-risk groups.

### Prediction of response to chemotherapy

To test the effectiveness of the model in the therapeutic intervention of COAD, we computed the IC_50_ of several chemotherapy-related medications that are routinely used from the STAD dataset (TCGA). The American Joint Committee on Cancer (AJCC) recommends utilizing chemotherapeutic medicines including mitomycin, paclitaxel, docetaxel, cisplatin, and doxorubicin for treating colon cancers. Using the Wilcoxon signed-rank test, a calculation was made to determine the IC_50_ difference that existed between the low- and high-risk groups. The results were displayed in the form of a box plot using the R packages "pRRophetic" and "ggplot2" [21].

### Statistical analysis

Correlation coefficients were calculated through pearson analysis. The prognostic significance was examined utilizing the Kaplan–Meier method, the Cox regression model, and log-rank tests. All analyses of statistical data were done in a two-sided manner and the threshold for statistical significance was fixed at *p*-value < 0.05. R (version 4.0.4) was utilized throughout the process to conduct all statistical analyses.

## Results

### Generating a weighted co-expression network and identifying key modules

**Fig. 1:**
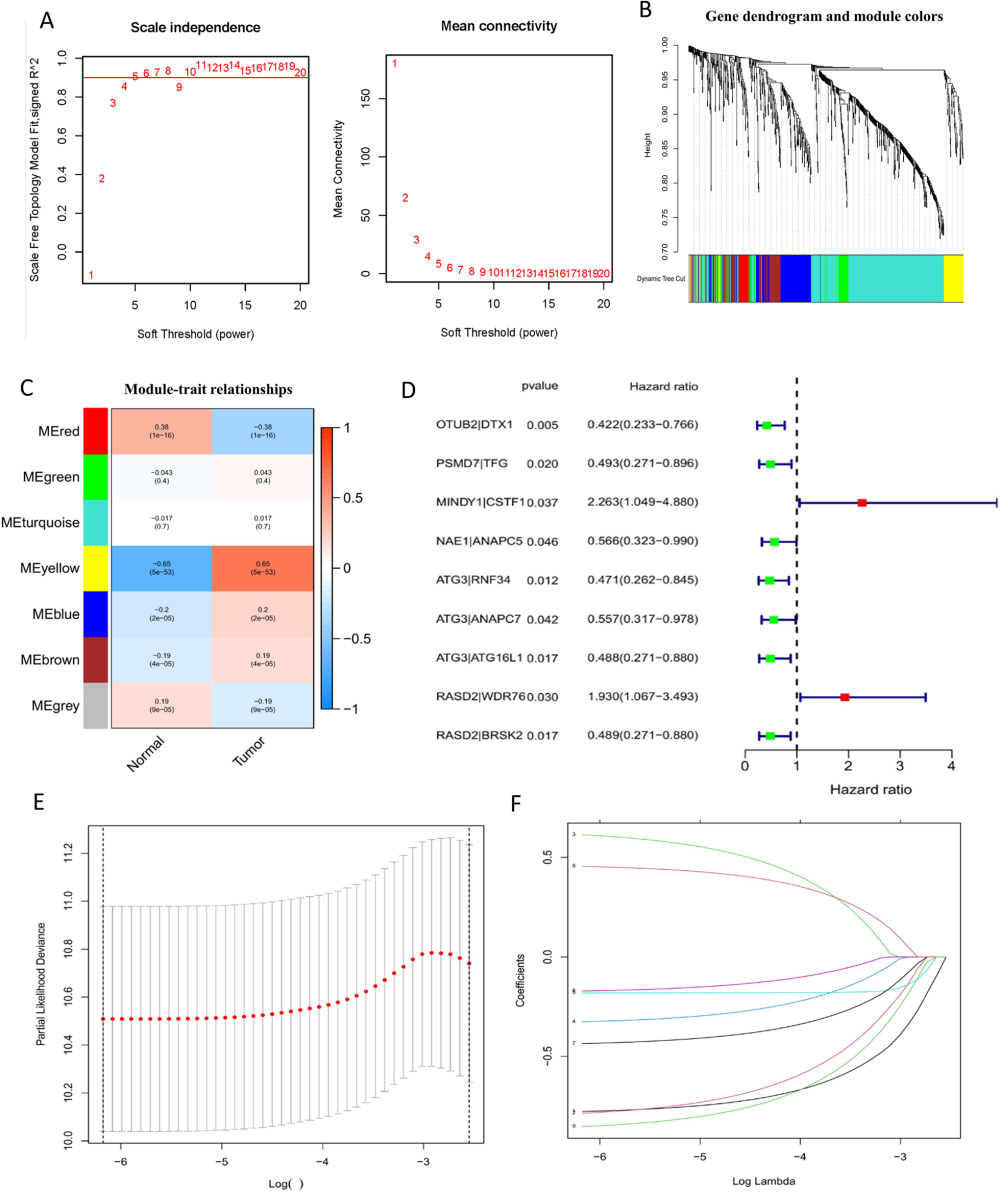
Identification and establishment of the URGPs signature in COAD. **A** Soft-thresholding power in WGCNA. **B** Tree of gene clusters. The dynamic tree cutting approach was applied to discover modules by separating the tree diagram at significant branch points. This was premised on an adjacency-based mismatch that was found in the hierarchical gene clustering chart. In the horizontal bar immediately below the tree diagram, various colors have been designated for each module. **C** Associations between modules and traits in normal and malignant tissues. The table is organized such that each row signifies a color module while each column signifies a clinical characteristic. The correlation coefficient between each module and clinical features and the *p*-value corresponding to that coefficient is shown by the numbers in each cell. **D** The forest plot depicting the prognostic-associated URGPs as determined by the univariate Cox proportional hazards regression model in COAD patients. **E** The calculation of penalties by one thousand rounds of cross-validation to get the optimal values for the parameters. **F** LASSO-Cox regression analysis was performed by computing the minimal criterion

We retrieved the expression matrices from the 367 samples that were included in the TCGA-COAD dataset following the preprocessing and quality evaluation of the data. In COAD patients, co-expression modules were discovered by generating co-expression networks from the TCGA-COAD utilizing the WGCNA system biology approach. In the current investigation, a scale-free network was constructed premised on a soft power of *β* = 5 (Fig. 1A), and 7 modules were derived from the TCGA-COAD dataset by means of the average linkage hierarchical clustering (Fig. 1B). In addition, we investigated the correlation of modules between clinical subsets (tumor and normal) and each module, which allowed us to discover key modules and generate the heatmaps of module–trait associations that are depicted in Fig. 1C. The MEyellow modules included within the TCGA-COAD (*r* = 0.65, *p* < 0.001) that were discovered as having the strongest correlation with tumor samples were chosen as clinically significant modules.

### Construction of URGPs prognostic signature

TCGA-COAD patients were classified at random into training (discovery cohort, *n* = 245) and test (validation cohort, *n* = 122) groups in a 2:1 ratio approximately. We started by screening 782 URGs and ended up with 514 URGPs. As per the findings of a univariate Cox regression analysis conducted on the training set, 9 URGPs were shown to be linked to OS, whereby, two URGPs (MINDY1|CSTF1, RASD2|WDR76) predicted unfavorable prognosis with a hazard ratio (HR) > 1 and seven URGPs (OTUB2|DTX1, PSMD7|TFG, NAE1|ANAPC5, ATG3|RNF34, ATG3|ANAPC7, ATG3|ATG16L1, RASD2|BRSK2) predicted favorable prognosis with a hazard ratio (HR) < 1 (Fig. 1D). To establish the URGPs signature, the LASSO-Cox proportional hazard regression method was utilized after the less-variable URGPs had been eliminated and clinical data had been analyzed in conjunction with it. Afterward, 6 URGPs comprised 11 URGs were utilized to generate the risk score model (Fig. 1E, F). In the last step of this process, we utilized the URGPs signature to assign a risk score to each patient within the discovery cohort. The computation of the risk score for the signature model was as follows = (Coefficient_URGP1_ × Score_URGP1_) + (Coefficient_URGP2_ × Score_URGP2_) + … + (Coefficient_URGP6_ × Score_URGP6_) (Additional file 3: Table S3).

### Validation of prognostic gene expression profiles

**Fig. 2:**
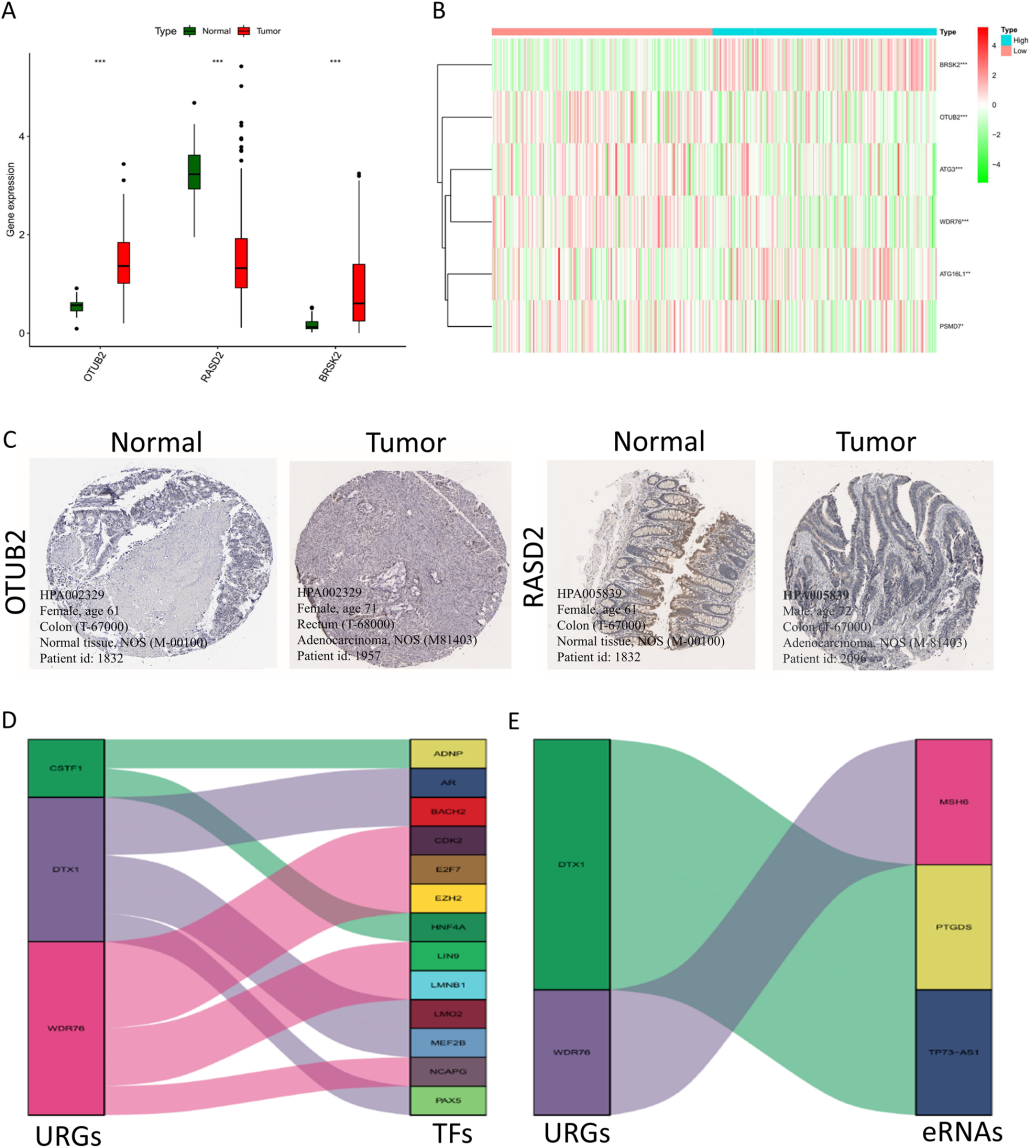
The expression of the genes involved in COAD patients' prognoses.** A** The expression profiles of the 3 genes in COAD and normal samples of the colon. Wilcoxon rank-sum tests were carried out to analyze the differences in the levels of gene expression that were observed between the tumor and the normal samples. ***p < 0.001. **B** A heat map of gene expression in the low- and high- risk groups. *p < 0.05, **p < 0.01, ***p < 0.001. **C** Immunohistochemistry images of 2 URGs (OTUB2, RASD2) in COAD and normal samples of the colon. **D** Sankey diagrams representing the potential regulatory relationships of URGs and TFs. **E** Sankey diagrams representing the potential regulatory relationships of URGs and eRNAs

To evaluate the function of the prognostic genes identified by the COAD-related predictive signature, we examined their gene expression levels in the TCGA database and their protein expression levels in the Human Protein Atlas (HPA) database. Three prognostic genes' expression levels were substantially changed in COAD compared to non-tumor tissues, as depicted in Fig. 2A (All *p*-values < 0.001), whereas the expression levels of six prognostic genes were considerably altered in the high-risk group in contrast with the low-risk group (Fig. 2B, all *p*-values < 0.05). Figure 2C depicts the prognostic genes’ characteristic immunohistochemistry images in tumor and normal samples, and the results illustrated considerable upregulation of the OTUB2 protein expression in COAD relative to normal tissue. On the contrary, decreased RASD2 protein expression was discovered in the COAD tissue. All of these factors support the results of our research on the differential expression of these genes. In addition, a Sankey diagram was used to illustrate the created TFs-hub genes network. This network was found to include 13 TFs as well as three hub genes (Fig. 2D). WDR76 served as the most important node among all the genes since it had the greatest degree; it was controlled by six TFs, comprising numerous essential genes related to tumors, such as CDK2, E2F7, and EZH2. Additionally, eRNAs are a subclass of lncRNAs that are known to exert a broad range of effects in human malignancies, here Pearson correlation analysis was implemented to predict eRNA–hub gene connections and investigate the underlying regulation mechanism by eRNAs in humans. As depicted in Fig. 2E, among the eRNA–hub gene interaction pairs, 3 eRNAs related to URGs were subsequently selected, and DTX1 had the greatest degree of connection; it was targeted by two eRNAs (PTGDS, TP73-AS1), indicating an association with human cancer that is increasing. Briefly, the TFs–hub gene interaction network, as well as the found eRNA–hub gene pairs, may shed light on the continued investigation of the molecular mechanisms underlying COAD.

### Verification of the URGPs signature and assessment of survival prediction

**Fig. 3:**
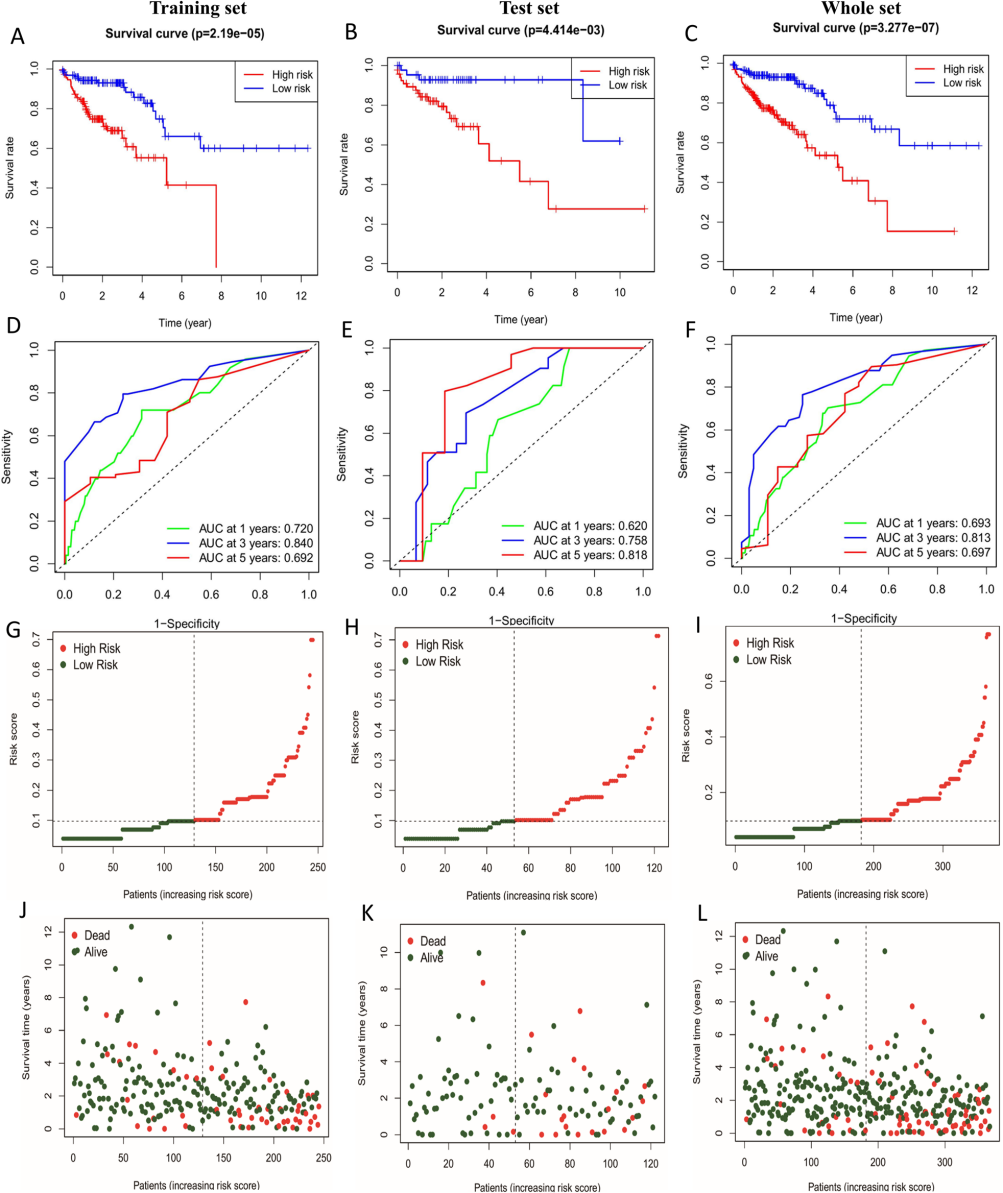
Assessment and confirmation of the predictive significance of the URGPs signature in COAD. **A** Plots representing the Kaplan–Meier overall survival data for the training group depending on the risk scores. **B** Plots of overall survival calculated using Kaplan–Meier for the test group as per risk scores. **C** The Kaplan–Meier plots show the overall survival rate in relation to the risk scores for the whole group. **D** In the training set, the ROC for overall survival was calculated. **E** ROC representing the overall survival rate of the test group. **F** ROC measures survival rates in the whole group. **G** The risk score distribution in the training group. **H** The risk score distribution in the test group. **I** The risk score distribution in the whole group. **J** Plot depicting the survival rates of patients belonging to the training group. **K** Survival plots of patients in the test group. **L** Plots showing the survival of patients in the whole group

We developed a predictive risk model for COAD patients using the 6-URGPs signature and estimated each patient’s risk score in the training set. In the training, validation, and TCGA sets, we discovered that the group with a high risk showed a worse prognosis in contrast with the group with a low risk premised on the median risk score (log-rank test *p*-value 0.05; Fig. 3A–C). In the training sets, the 1-, 3-, and 5-year AUC values were 0.720, 0.840, and 0.692, correspondingly (Fig. 3D). In addition, it is noteworthy that, for the 3-year AUC, the findings in each set indicated that all AUC values are > 0.75 (Fig. 3D–F), signifying that this model has a higher predictive value. These findings indicate that our 6-URGPs signature is an effective prognostic indicator for COAD.

### Prognostic significance of the URGPs signature and its link to clinical and pathological characteristics

**Fig. 4:**
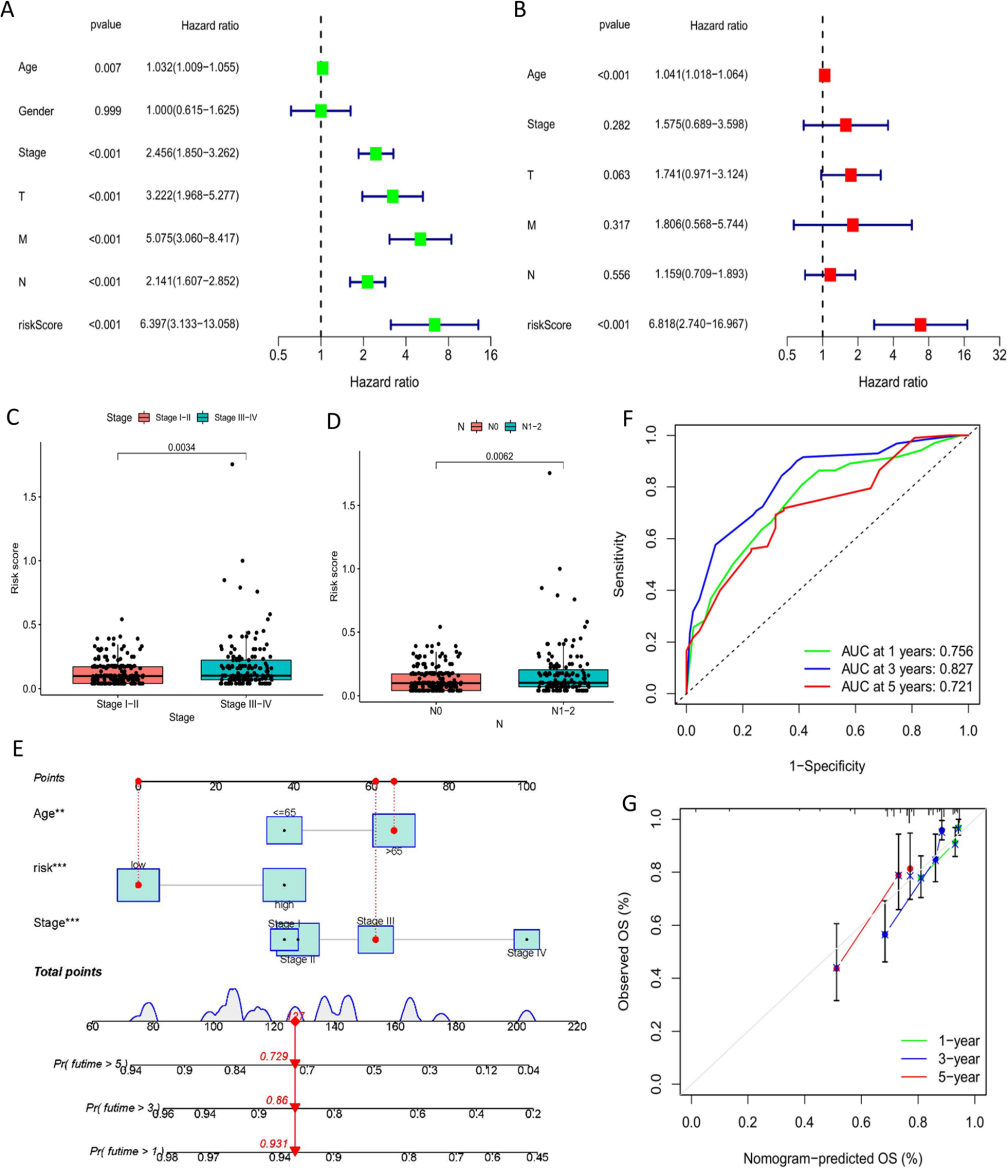
A nomogram that incorporates both clinical and pathological variables and the URGPs signature. **A** Univariate Cox regression examination of OS-related factors. **B** OS-related factors subjected to a multivariate Cox regression analysis. **C** Wilcoxon rank-sum test showed COAD risk scores were associated to clinical stage. **D** Wilcoxon rank-sum test showed COAD risk scores were connected to lymph node metastasis. **E** The nomogram for prognostic prediction in COAD. **F** The nomogram-based ROC curve analysis displays 1-, 3-, and 5-year OS and the corresponding AUC values for COAD patients from the TCGA cohort. **G** The calibration curve used to verify the predictive performance of the model

Figure 3G–L depicts the distribution of URGPs signature together with the relevant survival status depending on the risk curve. In each of the three datasets, as the risk score became larger, the number of fatalities and the percentage of patients who were at high risk got higher as well. We conducted univariate and multivariate Cox regression analyses of the URGPs signature and clinical and pathological parameters, comprising age, sex, stage, and TNM staging, to confirm the clinical utility of the URGPs signature. The findings showed that the URGP signature was a predictive factor for COAD that was independent of other variables (*p* < 0.001) (Fig. 4A, B). After that, we evaluated how the risk score varied depending on the clinicopathological characteristics that were included. It was found that the risk score distribution differed considerably when comparing stages I–II and III–IV, as well as N0 and N1–2 (*p* < 0.05, Fig. 4C, D). In summary, we completed a stratified survival analysis to ascertain the level of accuracy possessed by the URGPs signature in distinguishing distinct clinicopathological feature subgroups.

In addition, we combined the aforementioned clinicopathological markers with age, stage, and our 6-URGPs signature. Afterward, a nomogram was developed to objectively predict the COAD patients’ prognoses using the previously indicated clinicopathological criteria and the URGPs signature. Because gender did not contribute significantly to the predictive outcomes, it was excluded from the univariate analysis (*p* = 0.999). Then, age, stage, and URGPs signature were added to the nomogram to enhance the accuracy of the COAD prognostic prediction model (Fig. 4E). In the training group, the AUC for predicting 1 year, 3 years, and 5 years of overall survival in COAD was 0.756, 0.827, and 0.721, respectively, demonstrating strong discrimination (Fig. 4F). Figure 4G is a depiction of the calibration curve that was utilized to assess the predictive accuracy of the model, which demonstrates excellent congruence between the expected and actual results in the training datasets.

### Function and signaling pathways analysis of URGPs in the prognosis module

**Fig. 5:**
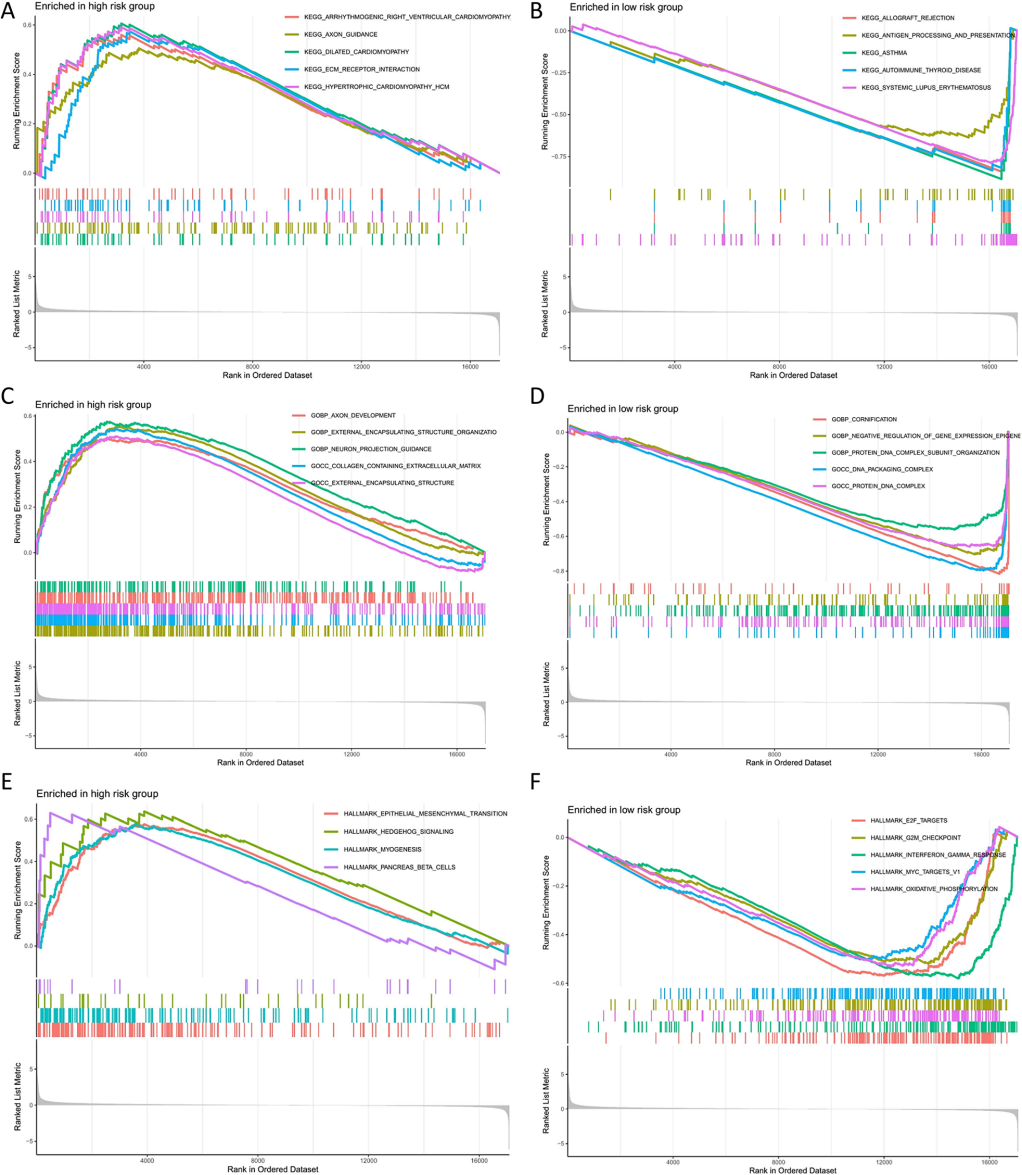
URGPs signature association with biological functions. **A**, **B** KEGG findings for the low- and high-risk groups. **C**, **D** Findings of the GOBP for the low- and high-risk groups. **E**, **F** GSEA reveals the hallmark pathways enriched in the low- and high-risk groups of the URGPs signatures in COAD

The ability of the model created by six URGPs to discriminate against patients with varied prognoses shows that patients having varying risk scores might be implicated in distinct critical pathways that contribute to disparities in prognosis. Premised on the aforementioned hypotheses, we conducted GSEA analyses on low- and high-risk patients, correspondingly, to verify major pathways in each group and the putative biological mechanisms of the URGPs signature involved in COAD progression. As per the findings of the KEGG enrichment, there are two distinct groups, each of which has its own signature pathways. Patients who have a low-risk score had higher upregulation levels regarding several pathways, including those related to autoimmune_thyroid_disease, asthma, antigen_processing_and_presentation, allograft_rejection, and systemic_lupus_erythematosus. Conversely, five pathways were shown to be upmodulated in the high-risk group, including, encompassing hypertrophic_cardiomyopathy_hcm, ecm_receptor_interaction, dilated_cardiomyopathy, axon_guidance, and arrhythmogenic_right_ventricular_cardiomyopathy (Fig. 5A, B). The enrichment of the low- and high-risk groups in the Gene Ontology biological process (GOBP) is displayed in Fig. 5C, D. We discovered an enrichment of the low-risk group in cornification, negative_regulation_ of_gene_expression_epigene, protein_dna_complex_subunit_organization, dna_packaging_complex, and protein_dna_complex. Conversely, the high-risk group was predominantly enriched in axon development, external_encapsulating_structure_organization, neuron_projection_guidance, and so on. The results of the most significant terms enriched by the hallmark gene set indicated that epithelial_mesenchymal_transition, hedgehog_signaling, myogenesis, and pancreas _beta _cells were activated by the high-risk group of the URGPs signature. Furthermore, gene sets such as myc_targets_v1, interferon_gamma_response, g2m_checkpoint, e2f_targets, and oxidative_phosphorylation were activated by the low-risk subgroup (Fig. 5E, F). These findings corroborated the above-mentioned findings that hub genes may have substantial interactions with one another, highlighting the critical function of hub genes in COAD tumor initiation and growth.

### Immune landscape in patients with COAD and immunotherapy analysis

**Fig. 6:**
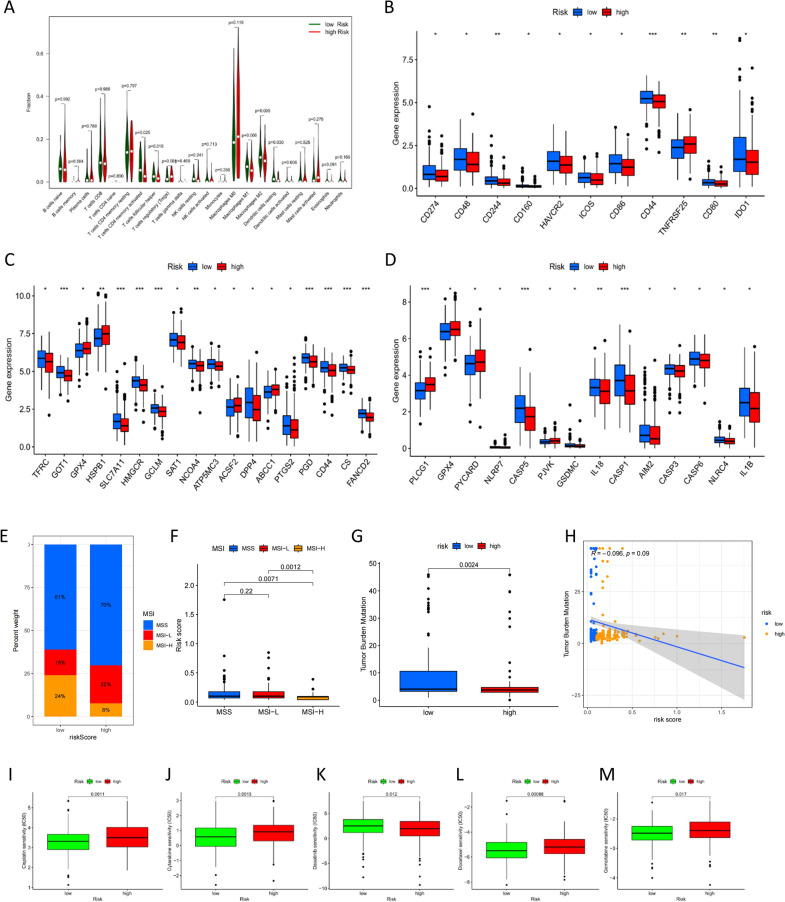
Correlation of URGPs risk score with MSI, TMB, and drug sensitivity in COAD patients. **A** The immunity infiltration difference between high- and low-risk scores. **B** The expression of immune checkpoint genes in the high- and low-risk groups. **C** Box plot depicting ferroptosis-related gene expression in two groups. **D** Box plot illustrating the expression of pyroptosis-related genes in two groups. **E**, **F** Relationship between the URGPs risk score and the MSI. **G** Comparison of TMB differences in the high- and low-risk groups. **H** Pearson correlation analysis of URGPs risk score and TMB. **I**–**M** The IC_50_ of 5 routinely used chemotherapy-related drugs (cisplatin, cytarabine, dasatinib, docetaxel and gemcitabine)

After that, we utilized CIBERSORT to study the association between the two risk groups and the immune infiltration by analyzing 22 distinct immune cell phenotypes from the training set. Figure 6A depicts the percentages of immune cell types in the training dataset's low- and high-risk groups. T cells CD4 memory-activated, follicular helper T cells, M1 and M2 macrophages, and resting dendritic cells were shown to be substantially elevated in low-risk patients. We discovered immune checkpoint inhibitors (ICIs)-related indicators by conducting a literature search and analyzing the therapeutic applications and advantages of these ICIs. We found that there were substantial differences between the two groups in the expression of a large number of immune checkpoints, indicating the two groups' varied immunological properties and immunotherapy effects (Fig. 6B).

In addition to this, it was discovered that the expressions of the majority of genes related to ferroptosis were higher in the low-risk group than they were in the high-risk group (Fig. 6C), and patients in the low-risk group also displayed a higher level of pyroptosis-related gene expressions than patients in the high-risk group did (Fig. 6D). All of these data reinforced the finding that COAD patients who were in the low-risk group possessed an immune "hot" profile and would benefit more from immunotherapeutic treatments. In other words, the URGPs signature may accurately predict the immunological features of human COAD.

Changes in MSI and TMB can have an impact on the effectiveness of immunotherapy. As a result of our investigation, we found that MSS and MSI-L were more likely to occur in the high-risk group compared with the low-risk group, but the proportion of MSI-H was significantly higher in the low-risk group than in the high-risk group (24% vs. 8%; Fig. 6E). The MSI-H patients exhibited lower risk scores than the MSI-L and MSS patients (*p* < 0.01; Fig. 6F). Treatment with immunotherapy was particularly beneficial for patients with low risk. We compared the TMB of high- and low-risk groups and discovered statistically significant differences (*p* < 0.01; Fig. 6G). Furthermore, the TMB was inversely associated to the URGPs risk score (*p* = 0.09; Fig. 6H).

### Analysis of the association between risk assessment models and chemotherapeutics

Besides the checkpoint blockade treatment, we established the connection that exists between risk assessment models and the effectiveness of conventional chemotherapeutic drugs in the treatment of COAD. With the aid of the pRRophetic algorithm, we discovered a link between high-risk score and decreased half-inhibitory concentration (IC_50_) of dasatinib (*p* = 0.012, Fig. 6K), and elevated IC_50_ for cisplatin (*p* = 0.0011, Fig. 6I), cytarabine (*p* = 0.0013, Fig. 6J), docetaxel (*p* = 0.00088, Fig. 6L) and gemcitabine (*p* = 0.017, Fig. 6M). According to the findings provided above, this risk-adjusted model could be a feasible predictor of patient sensitivity to chemotherapy.

## Discussion

The death rate associated with COAD is among the highest of all malignancies affecting the digestive system. It is more prevalent in guys older than 40. Nonetheless, early detection of COAD is extremely challenging, and the majority of patients who were identified with COAD have progressed malignancy, which resulted in a poor prognosis. It is possible to remarkably improve the COAD patients' prognoses through early detection and treatment, which also reduces the patients' financial burden and enhances their quality of life. Due to the advancement of RNA sequencing technologies, several molecular markers have been described as predictors of prognosis and therapy success in colorectal cancer (CRC). Recent research has found that epigenetic alterations, such as gene malfunction and aberrant expression, are implicated in the onset and advancement of numerous human cancers. Some research has looked into whether core epigenetic modification-related genes could be employed as cancer biomarkers. UB is the founding member of the structurally conserved protein family that is responsible for the modulation of a wide variety of functions in eukaryotic cells, particularly, substrate activation or inactivation, protein activation, and protein–protein interactions [22]. Protein ubiquitination and deubiquitination play a critical role in protein stability, localization, and signal pathway regulation, and disruptions in protein homeostasis can lead to a range of illnesses, including neurological disorders, autoimmune disorders, and cancers. It has been discovered that aberrant E3s expression and DUBs might impact human cancers by altering the activities of tumor-associated proteins. RING-finger E3 ubiquitin ligase MDM2 is primarily responsible for the monoubiquitination of p53, which influences p53 activity by modulating p53 localization and transcription functions. Numerous E4 ubiquitin ligases (E4s), which are responsible for the extension of these monoubiquitin chains, have also been identified [23, 24]. Nonetheless, only a limited number of ubiquitin molecules have been investigated in-depth, with the majority of studies focusing on the function of specific genes. Using expression profile information, few research reports have comprehensively investigated the molecular features and prognostic potential of URGs. This is the first research that we know that utilizes URGPs to design a risk model for predicting COAD patients' prognoses.

In this work, several URGs were identified by conducting an analysis of the COAD dataset included within the TCGA database. Following that, the gene modules associated with the onset and progression of COAD were filtered by WGCNA in a systematic manner. As an unsupervised algorithm, WGCNA can build a relationship between gene expression and clinical traits [25]. Rather than focusing solely on differentially expressed genes, WGCNA identifies gene sets of interest and performs extensive association analysis with phenotypes, transforming the problem of multiple hypothesis testing corrections by transforming the correlation of thousands of genes with phenotypes into the association of several gene sets. Then an in-depth examination of the genes contained within the module was performed. Finding the optimum approach still poses challenges despite the fact that numerous research have employed various machine learning techniques to choose the appropriate variables. In this investigation, we employed the most traditional, widely applied technique [26, 27].The LASSO regression technique is a penalized form of regression that reduces the size of certain coefficients to produce a more accurate model by the construction of a penalty function. It is an estimator that is biased because it processed data that have complicated collinearity. However, it is frequently employed in high-dimensional regression and may help compensate for the deficiencies of univariate Cox regression analysis [28, 29]. Following additional processing using LASSO regression, 6 URGPs linked to the advancement of the tumor were ultimately found. These gene pairs were subsequently subjected to the multivariate Cox regression to establish a risk model for predicting the patients' prognoses. Additionally, it was discovered through ROC curve validation of the model's effectiveness that its prediction capacity of COAD patients' survival over 1, 3, and 5 years in the dataset was moderately accurate. In summary, both the univariate and the final multivariate Cox regression analyses illustrated that the risk model independently functioned as prognostic indicators. We observed that the established nomogram premised on this model performed well when we calibrated it. Numerous earlier research reports have constructed risk models that could accurately anticipate the COAD prognosis, and the majority of these models incorporate multiple functional gene sets. For instance, Chen et al. developed and validated a COAD predictive risk model using a total of 8 lncRNAs that are associated with endoplasmic reticulum (ER) stress [30]. Rong H and colleagues discovered a novel genetic signature that is associated with the invasion of COAD. This study created a risk model for forecasting COAD prognoses as well as a nomogram based on this model to collectively examine the prognosis of patients with TNM staging, providing insights and guidance for fundamental COAD research [31]. Many of these studies have been reported in other types of cancer, such as lung adenocarcinoma (LUAD) [32, 33].

In our study, 6 URGPs were incorporated in our signature, of which OTUB2|DTX1, PSMD7|TFG, ATG3|ATG16L1, RASD2|BRSK2 were determined to be risk protective markers. The remaining 2 pairs (MINDY1|CSTF1, RASD2|WDR76) were determined to be risk-related indicators. Previous research has established that several genes within each of these gene pairs perform an integral function in the advancement of COAD. One study suggests that deubiquitinase OTUB2 exacerbates colorectal cancer growth by increasing PKM2 activity and glycolysis [34]. Lower levels of DTX1 could promote breast cancer (BC) cell proliferation and migration and are associated with advanced BC [35]. While MINDY1, a member of the motif interacting with Ub-containing novel DUB family, has been identified as a potential estrogen receptor α (ERα) deubiquitylase in BC. High MINDY1 expression was linked to a poor prognosis of BC [36]. In LUAD, PSMD7 expression was linked to not only tumor laterality, but also lymph node invasion. In LUAD patients, an elevated level of PSMD7 was linked to the unfavorable OS and disease-free survival, and PSMD7 silencing considerably attenuated the proliferative ability of cells and triggered the G0/G1-phase cell cycle arrest, cell senescence, and apoptosis [37]. ATG3 and ATG16L1 were key players with important roles in different stages of autophagy. The researchers Huang et al. found that overexpression of ATG3, which was caused by downregulation of miR-435-5p, increases proliferative and invasive capacities in CRC via an autophagy-dependent process [38]. The results from Florin et al. suggested that ATG16L1 T300A polymorphism may be associated with gastric carcinogenesis [39]. RASD2 encodes a Ras-related GTP-binding protein and involves in the development and metastasis of Uveal melanoma [40]. In CRC, RAS stabilization is a critical event for hyperactivation of Wnt/-catenin signaling and cancer stem cell activation. WDR76 has been shown to destabilize RAS and serves as a tumor inhibitor in CRC by suppressing cancer stem cell activation [41]. BRSK2, which belongs to the serine/threonine-protein kinase of the AMPK family, was recognized to be a risk factor for pancreatic ductal adenocarcinoma (PDAC). BRSK2 was induced by nutritional deprivation in PDAC cells, which inhibited TORC1 activity through tuberous sclerosis complex 2 (TSC2) phosphorylation [41].

This URGPs signature exhibited an excellent diagnostic capacity and may be utilized to distinguish COAD patients with an unfortunate prognosis, as determined by the survival and ROC curve analyses in the TCGA dataset. Additionally, the URGPs may predicted the OS of COAD patients in various clinical and pathological stratifications, and results showed that the signature was substantially linked to advanced clinical and pathologic stage. In the end, a nomogram was designed to fulfill the need for an easy-to-understand and practical scoring system, as well as to facilitate clinical decision-making. In addition, the gene functional enrichment analysis has shown that the URGPs are implicated in the onset and progression of COAD by engaging in a range of critical biological processes. Immunotherapy is a field that is now undergoing intensive research and innovation in COAD. Recent research has revealed that although TME performs an instrumental function in immunotherapy, the particular processes implicated are not yet completely understood [43]. Therefore, it is vital to conduct further studies on the involvement of the TME to increase the immunotherapeutic efficacy. COAD's TME consists of stromal cellular components, tumor cells, and immune cells. There is significant evidence that immune cells present in the TME impact carcinogenesis. The dysfunction of immune cells can have a range of outcomes, including those that are antitumorigenic or protumorigenic.

In this investigation, we adopted the CIBERSORT algorithms to ascertain the infiltration levels of immune cells correspondingly. In addition, we discovered that COAD samples having low-risk scores were linked to greater infiltration levels of follicular helper T cell, and CD4 memory activated T cell, as well as higher M1 macrophages. Immune checkpoint molecules, which function as inhibitory receptors, are detected on the immune cells' surface and are responsible for regulating the immune response. According to a growing body of research, the expression of immune checkpoint biological markers may serve as a positive predictive indicator for the effectiveness of immunotherapeutic interventions. As a result, we subsequently evaluated the relationship of the URGPs signature with the expression of immune checkpoint biological markers and discovered that COAD patients who were in the low-risk group exhibited elevated expression levels of immune checkpoint biological markers in contrast with those who were in the high-risk group. To summarize, "immune active" was the term used to describe the immunological landscape of low-risk tumors, which were characterized by a significant infiltration of immune cells. This was accomplished by cleaving UB that was attached to substrates or contained inside UB chains. In the regulation of key cellular functions, DUBs serve critical functions, functioning either as switches that eliminate UB signals or as rheostats to fine-tune the degree and kind of ubiquitylation that takes place. Furthermore, the immune milieu of high-risk cancers was described as "immune inactive" with limited infiltration levels. This suggests that the current URGPs signature may accurately predict the immunological aspects of COAD, and patients who are within the low-risk group have a greater likelihood of gaining benefit from anti-tumor immunotherapeutic intervention as opposed to those within the high-risk group.

According to our current knowledge, a predictive model that is premised on URGPs and the related nomogram in COAD are yet to be investigated. This model showed an excellent prediction accuracy, and it might help distinguish patients who have a high recurrence risk and choose the appropriate treatment. Nevertheless, there are a few drawbacks to consider. Firstly, this work is a retrospective examination of publicly available datasets, which makes it prone to bias. As a result, a large-sample prospective clinical investigation needs to be carried out to evaluate the robustness of the signature model. Secondly, further research needs to be done to better understand the specific molecular processes and biological activities of the URGs. Lastly, the methodology that is dependent on gene-level prognostic features to anticipate the cost of samples is expensive, and there is significant clinical promotion resistance.

## Conclusions

Using bioinformatics approaches, we thoroughly evaluated the prognostic value of URGPs and developed a predictive model for COAD. The nomogram integrating clinicopathological data and the proposed URGPs signature can accurately predict COAD patients’ prognoses and aid doctors in the selection of individualized treatments. Additionally, it describes the connection between our signature and the sensitivity of immunological checkpoints as well as targeted medicines. These findings will aid in enhancing the predictive value of conventional clinical detection, which may be used to evaluate COAD clinical outcomes and contribute to precision medicine. Prospective research is required to confirm its validity.

## Supplementary Information

**Additional file 1: Table S1**. Expressing characteristics of patients with COAD in TCGA database.

**Additional file 2: Table S2**. 807 URGs.

**Additional file 3: Table S3.** The list of gene pairs and corresponding coefficient.

## Data Availability

The original contributions made in the research are outlined in the paper and Additional files. Corresponding authors may be contacted for any more questions.

## Abbreviations

COAD: Colon adenocarcinoma
PD-L1: Programmed death-ligand 1
PD-1: Programmed cell death protein-1
CTLA4: Cytotoxic T lymphocyte-associated antigen-4
ICB: Immune checkpoint blockade
ICIs: Immune checkpoint inhibitors
UB/UBL: Ubiquitin and ubiquitin-like
E1s: Ubiquitin-activating enzymes
E2s: Ubiquitin-conjugating enzymes
E3s: Ubiquitin-protein ligases
E4s: E4 ubiquitin ligases
DUBs: Deubiquitinases
ULDs: Ubiquitin-like domains
UBDs: Ubiquitin-binding domains
TCGA: The Cancer Genome Atlas
URGs: Ubiquitin-related genes
URGPs: Ubiquitin-related gene pairs
FPKM: Fragments per kilobase million
LASSO: Least absolute shrinkage, and selection operator
ROC: Receiver operating characteristics
WGCNA: Weighted gene co-expression network analysis
GSEA: Gene Set Enrichment Analysis
KEGG: Kyoto Encyclopedia of Genes and Genomes
AJCC: American Joint Committee on Cancer
OS: Overall survival
AUC: Area under the curve
C-index: Concordance index
TFs: Transcription factors
eRNAs: Enhancer RNAs
TME: Tumor microenvironment
MSI: Microsatellite instability
TMB: Tumor mutation burden
MMR-D: DNA mismatch repair
MSI-H: Microsatellite instability
CRC: Colorectal cancer
BC: Breast cancer
ER: Endoplasmic reticulum
LUAD: Lung adenocarcinoma
MINDY: Motif interacting with Ub containing novel DUB family
PDAC: Pancreatic ductal adenocarcinoma
TSC2: Tuberous sclerosis complex 2

## References

[1] Kriegsmann M, Longuespee R, Wandernoth P, Mohanu C, Lisenko K, Weichert W, Warth A, Dienemann H, De Pauw E, Katzenberger T, Aust D, Baretton G, Kriegsmann J, Casadonte R (2017). Typing of colon and lung adenocarcinoma by high throughput imaging mass spectrometry. Biochim Biophys Acta Proteins Proteom.

[2] Lombardi L, Morelli F, Cinieri S, Santini D, Silvestris N, Fazio N, Orlando L, Tonini G, Colucci G, Maiello E (2010). Adjuvant colon cancer chemotherapy: where we are and where we'll go. Cancer Treat Rev.

[3] Cass AW, Million RR, Pfaff WW (1976). Patterns of recurrence following surgery alone for adenocarcinoma of the colon and rectum. Cancer AM Cancer Soc.

[4] Keum N, Giovannucci E (2019). Global burden of colorectal cancer: emerging trends, risk factors and prevention strategies. Nat Rev Gastroenterol Hepatol.

[5] Kanani A, Veen T, Soreide K (2021). Neoadjuvant immunotherapy in primary and metastatic colorectal cancer. Br J Surg.

[6] Swatek KN, Komander D (2016). Ubiquitin modifications. Cell Res.

[7] Pickart CM (2001). Ubiquitin enters the new millennium. Mol Cell.

[8] Meng Y, Qiu L, Zhang S, Han J (2021). The emerging roles of E3 ubiquitin ligases in ovarian cancer chemoresistance. Cancer Drug Resist.

[9] Heride C, Urbe S, Clague MJ (2014). Ubiquitin code assembly and disassembly. Curr Biol.

[10] Park HB, Baek KH (2022). E3 ligases and deubiquitinating enzymes regulating the MAPK signaling pathway in cancers. Biochim Biophys Acta Rev Cancer.

[11] Buchberger A (2002). From UBA to UBX: new words in the ubiquitin vocabulary. Trends Cell Biol.

[12] Popovic D, Vucic D, Dikic I (2014). Ubiquitination in disease pathogenesis and treatment. Nat Med.

[13] Mortazavi A, Williams BA, McCue K, Schaeffer L, Wold B (2008). Mapping and quantifying mammalian transcriptomes by RNA-Seq. Nat Methods.

[14] Zhou J, Xu Y, Lin S, Guo Y, Deng W, Zhang Y, Guo A, Xue Y (2018). iUUCD 2.0: an update with rich annotations for ubiquitin and ubiquitin-like conjugations. Nucleic Acids Res.

[15] Ma Z, Zhong P, Yue P, Sun Z (2022). Uncovering of key pathways and miRNAs for intracranial aneurysm based on weighted gene co-expression network analysis. Eur Neurol.

[16] Cao K, Liu M, Ma K, Jiang X, Ma J, Zhu J (2022). Prediction of prognosis and immunotherapy response with a robust immune-related lncRNA pair signature in lung adenocarcinoma. Cancer Immunol Immunother.

[17] Huang CW, Syed-Abdul S, Jian WS, Iqbal U, Nguyen PA, Lee P, Lin SH, Hsu WD, Wu MS, Wang CF, Ma KL, Li YC (2015). A novel tool for visualizing chronic kidney disease associated polymorbidity: a 13-year cohort study in Taiwan. J Am Med Inform Assoc.

[18] Subramanian A, Tamayo P, Mootha VK, Mukherjee S, Ebert BL, Gillette MA, Paulovich A, Pomeroy SL, Golub TR, Lander ES, Mesirov JP (2005). Gene set enrichment analysis: a knowledge-based approach for interpreting genome-wide expression profiles. Proc Natl Acad Sci USA.

[19] Kanehisa M, Sato Y, Morishima K (2016). BlastKOALA and GhostKOALA: KEGG tools for functional characterization of genome and metagenome sequences. J Mol Biol.

[20] Newman AM, Liu CL, Green MR, Gentles AJ, Feng W, Xu Y, Hoang CD, Diehn M, Alizadeh AA (2015). Robust enumeration of cell subsets from tissue expression profiles. Nat Methods.

[21] Geeleher P, Cox N, Huang RS (2014). pRRophetic: an R package for prediction of clinical chemotherapeutic response from tumor gene expression levels. PLoS ONE.

[22] Pickart CM, Eddins MJ (2004). Ubiquitin: structures, functions, mechanisms. Biochim Biophys Acta.

[23] Love IM, Shi D, Grossman SR (2013). p53 Ubiquitination and proteasomal degradation. Methods Mol Biol.

[24] Li YC, Cai SW, Shu YB, Chen MW, Shi Z (2022). USP15 in cancer and other diseases: from diverse functions to therapeutic targets. Biomedicines.

[25] Langfelder P, Horvath S (2008). WGCNA: an R package for weighted correlation network analysis. BMC Bioinformatics.

[26] Lai G, Zhong X, Liu H, Deng J, Li K, Xie B (2022). Development of a Hallmark pathway-related gene signature associated with immune response for lower grade gliomas. Int J Mol Sci.

[27] Lai G, Liu H, Deng J, Li K, Xie B (2022). A novel 3-gene signature for identifying COVID-19 patients based on bioinformatics and machine learning. Genes (Basel).

[28] Tibshirani R (1997). The lasso method for variable selection in the Cox model. Stat Med.

[29] Wang W, Liu W. PCLasso: a protein complex-based, group lasso-Cox model for accurate prognosis and risk protein complex discovery. Brief Bioinform. 2021; 22. 10.1093/bib/bbab212 34086850

[30] Chen X, Gao K, Xiang Z, Zhang Y, Peng X (2022). Identification and validation of an endoplasmic reticulum stress-related lncRNA signature for colon adenocarcinoma patients. Int J Gen Med.

[31] Rong H, Li Y, Hu S, Gao L, Yi T, Xie Y, Cai P, Li J, Dai X, Ye M, Liao Q (2022). Prognostic signatures and potential pathogenesis of eRNAs-related genes in colon adenocarcinoma. Mol Carcinog.

[32] Jiang F, Hu Y, Liu X, Wang M, Wu C (2022). Methylation pattern mediated by m(6)A regulator and tumor microenvironment invasion in lung adenocarcinoma. Oxid Med Cell Longev.

[33] Guo CR, Mao Y, Jiang F, Juan CX, Zhou GP, Li N (2022). Computational detection of a genome instability-derived lncRNA signature for predicting the clinical outcome of lung adenocarcinoma. Cancer Med.

[34] Yu S, Zang W, Qiu Y, Liao L, Zheng X (2022). Deubiquitinase OTUB2 exacerbates the progression of colorectal cancer by promoting PKM2 activity and glycolysis. Oncogene.

[35] Liu X, Xian Y, Xu H, Hu M, Che K, Liu X, Wang H (2021). The associations between Deltex1 and clinical characteristics of breast cancer. Gland Surg.

[36] Tang J, Luo Y, Long G, Zhou L (2022). Correction to: MINDY1 promotes breast cancer cell proliferation by stabilizing estrogen receptor alpha. Cell Death Dis.

[37] Xu X, Xuan X, Zhang J, Xu H, Yang X, Zhang L, Zhao Y, Xu H, Li D (2021). PSMD7 downregulation suppresses lung cancer progression by regulating the p53 pathway. J Cancer.

[38] Huang W, Zeng C, Hu S, Wang L, Liu J (2019). ATG3, a target of miR-431-5p, promotes proliferation and invasion of colon cancer via promoting autophagy. Cancer Manag Res.

[39] Burada F, Ciurea ME, Nicoli R, Streata I, Vilcea ID, Rogoveanu I, Ioana M (2016). ATG16L1 T300A polymorphism is correlated with gastric cancer susceptibility. Pathol Oncol Res.

[40] Xie M, Xin C (2022). RASD2 promotes the development and metastasis of uveal melanoma via enhancing glycolysis. Biochem Biophys Res Commun.

[41] Ro EJ, Cho YH, Jeong WJ, Park JC, Min DS, Choi KY (2019). WDR76 degrades RAS and suppresses cancer stem cell activation in colorectal cancer. Cell Commun Signal.

[42] Saiyin H, Na N, Han X, Fang Y, Wu Y, Lou W, Yang X (2017). BRSK2 induced by nutrient deprivation promotes Akt activity in pancreatic cancer via downregulation of mTOR activity. Oncotarget.

[43] Lin A, Zhang J, Luo P (2020). Crosstalk between the MSI status and tumor microenvironment in colorectal cancer. Front Immunol.

